# Role of hyaluronic acid intrauterine injection in the prevention of Asherman's syndrome in women undergoing uterine septum resection: An RCT

**DOI:** 10.18502/ijrm.v19i4.9060

**Published:** 2021-04-22

**Authors:** Seiede Zahra Ghanadzadeh Tafti, Atiye Javaheri, Razieh Dehghani Firoozabadi, Samane Kabirpour Ashkezar, Hossein Falahzadeh Abarghouei

**Affiliations:** ^1^Department of Obstetrics and Gynecology, Faculty of Medicine, Shahid Sadoughi University of Medical Sciences, Yazd, Iran.; ^2^Research and Clinical Center for Infertility, Yazd Reproductive Sciences Institute, Shahid Sadoughi University of Medical Sciences, Yazd, Iran.; ^3^Department of Biostatistics, Faculty of Health, Shahid Sadoughi University of Medical Sciences, Yazd, Iran.

**Keywords:** Asherman's syndrome, Septum, Uterine, Hyaluronic acid, Resection.

## Abstract

**Background:**

Adhesion due to intrauterine surgery such as septal resection associated with damaged endometrium can increase the risk of Asherman's syndrome. The main goal of treatment in this syndrome is to repair the damaged endometrium for creating a physiological pregnancy.

**Objective:**

To investigate the effect of intrauterine injection of hyaluronic acid on the prevention of Asherman's syndrome in women undergoing uterine septum resection.

**Materials and Methods:**

In this double-blind randomized clinical trial, 65 women undergoing the uterine septum resection were divided into two groups; the case group (n = 34) and the control group (n = 31). Immediately after the septal resection with a resectoscope, 1cc of hyaluronic acid gel in the case group and 1cc normal saline solution as a placebo in the control group was injected into the uterine cavity. After two months, existence of intrauterine adhesions in the both groups was examined by the hysteroscope and assessment of menstrual patterns, according to the American Society for Reproductive Medicine criteria.

**Results:**

Our results showed that after intervention, the incidence of Asherman's syndrome in the control group was higher than the case group (p = 0.012). In the case group, only four women had poor adhesion (Asherman's syndrome) at the end of the study, while the rest of them were free of any adhesions in the uterine cavity. In the control group, however, only 19 were free of intrauterine adhesions and 12 had mild symptoms.

**Conclusion:**

The results of the study exhibited the hyaluronic acid capacity to reduce the risk of Asherman's syndrome in women with endometrial damage following a septal resection surgery.

## 1. Introduction

Asherman's syndrome, or intrauterine adhesion, is an acquired disease involving intrauterine adhesions that occur usually as a result of pregnancy, childbirth, infection, or uterine surgery such as myomectomy or correction of uterine abnormalities (e.g., intrauterine septum or bicornuate uterus) (1-3). If the Asherman's syndrome causes infertility and amenorrhea, treatment is necessary. In such cases, surgical treatment is performed by cutting and removing the adhesions using hysteroscopic and laparoscopic methods. Complications of hysteroscopic surgery include bleeding, uterine perforation, and pelvic infection. However, in some patients, infertility is not treated even after the treatment of Asherman's syndrome (intrauterine adhesions) (4, 5). The main goal of treatment in this syndrome is to repair the damaged endometrium as well as create a physiological condition suitable for pregnancy. Recently, drugs containing 100% hyaluronic acid have been used to treat adhesions.

Hyaluronic acid is an organic compound also known as hyaluronan. Polymeric hyaluronic acid is composed of D-glucuronic acid and N-acetyl glucosamine. Hyaluronic acid chains can be composed of 25,000 units or more, and the molecular weight of these chains varies between 5,000 and 20 million daltons. Hyaluronic acid is made by an enzyme called hyaluronan synthase. At least three types of hyaluronan synthetic enzyme (HAS) are available to humans: HAS1, HAS2, and HAS3. Types 1 and 2 produce high molecular-weight hyaluronic acids while type three generates light hyaluronic acids. Hyaluronic acid is degenerated by an enzyme called hyaluronidase (6, 7). Its anti-adhesive effect depends on the molecular weight and concentration of the product. The gel containing 100% hyaluronic acid, which is very sticky and acts as a mechanical barrier in the uterine cavity, prevents adhesion. Adhesions usually develop from the third to the fifth day after the surgery and the hyaluronic acid composition remains in place for seven days after which it is completely absorbed. Several studies have investigated the effect of hyaluronic acid compounds in preventing adhesions and the development of Asherman's syndrome following manipulation of the uterine cavity in animal and human models and have reported the capacity of the hyaluronic acid gel in keeping the uterine walls in place for 72 hr. Hysteroscopic studies have also shown a statistically significant decrease in the intrauterine adhesions a few months following the surgery (8-15).

Due to the features mentioned for this compound, this study was designed and conducted to investigate the use of hyaluronic acid in the prevention of Asherman's syndrome in women undergoing uterine septum resection.

## 2. Materials and Methods

This randomized double-blind clinical trial was performed on women, aged 15-45 yr, that were referred to the Shahid Sadoughi Hospital in Yazd, Iran for septal resection surgery between November and December 2019. Participants were primarily divided into two groups of case (n = 34) and control (n = 31) using random number tables. Our exclusion criteria comprised body mass index > 35 kg/m${}^{2}$, menopause, pregnancy, history of cervical cancer, coagulation disorders, and severe pelvic infections.

The sample size was estimated to be 80 (40 in each group), considering the significance level of 5%, the power of 80%, based on a previous similar article (16), and to reach the difference of 20% for the degree of adhesion in the two groups.

$$n=\frac{{\left(Z_{\left(1-\frac{\alpha }{2}\right)}+Z_{1-\beta }\right)}^{2}\left[P_{A}\left(1-P_{A}\right)+P_{B}\left(1-P_{B}\right)\right]}{{\left(P_{A}-P_{B}\right)}^{2}}$$

All participants underwent uterine septal resection using a rectoscope. In the case group, 1${}^{cc}$ of hyaluronic acid gel (Humedix Co., Korea) and in the controls, 1${}^{cc}$ of normal saline solution as a placebo was injected into the uterine cavity, immediately after surgery. Hyaluronic acid gels and normal saline were prepared and coded in similar syringes with the same volume, and one Obstetrics and Gynecology resident performed the injections. All participants, project executives, and researchers were blinded to the type of solution and group classification. Two groups then underwent hormone therapy with estrogen conjugate 1.25 mg/day and medroxyprogesterone 5 mg/day orally from day 14 to 24 of the menstrual cycle for two months. Two months after the intervention, intrauterine adhesion score was examined through hysteroscopy and clinical signs (menstrual pattern) were classified into mild, moderate, and severe types according to the American Society for Reproductive Medicine (ASRM) criteria as following:

(i) Mild: Involvement of < 1/3 of the uterine cavity and the presence of delicate adhesions with a regular menstrual pattern.

(ii) Moderate: Involvement of 1/3-2/3 of the uterine cavity with delicate-to-moderate adhesions with most hypomenorrhea.

(iii) Severe: Involvement of > 2/3 of the uterine cavity and the presence of thick bands of adhesion with amenorrhea.

In the beginning of the study, basic characteristics of the participants including age, gravity, number of live births, abortion, stillbirth, and the menstrual pattern were recorded in the data collection form. Finally, the number of menstrual days and volume of menstrual bleeding before and after the intervention, as well as the incidence and severity of Asherman's syndrome was investigated and compared between the groups in terms of adhesion score.

### Ethical considerations

All participants were orally informed about the purpose of this study and the procedure thereof, and a written informed consent was obtained from each one of them prior to the study. In addition, the research proposal was reviewed and approved by the Ethics Committee of Shahid Sadoughi University of Medical Sciences, Yazd, Iran (Code: IR.SSU.MEDICINE.REC.1398.201).

### Statistical analysis

Data were entered into the SPSS software, version 22 (Statistical Package for the Social Sciences, version 22.0, SPSS Inc., Chicago, Illinois, USA). The mean ± standard deviation, and variance of the variables were calculated and compared using analytical tests including Student's *t* test and Chi-square test between the two groups. The P-value < 0.05 was considered as significant.

## 3. Results

**Figure 1:**
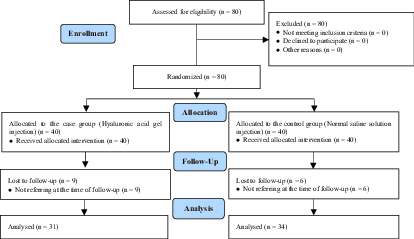
Consort flowchart of the studied participants.

Of the 80 participants who initially entered into the study according to the mentioned inclusion and exclusion criteria, 9 from the case group and 6 from the control group, due to not referring at the time of follow-up were lost subsequently and, therefore, 65 women were included in the final analysis (34 in the case group and 31 in the control group) (Figure 1). No significant difference was observed between the two groups in terms of age, gravidity, number of miscarriages, and stillbirths (Table I).

Additionally, the two groups were not significantly different as for menstrual patterns (number of days and volume of menstrual bleeding) before and after the intervention. The menstrual patterns of the participants in two groups were as follows: 21 (61.8%) women had a regular menstruation pattern in both groups, while 13 (38.2%) women in the case group and 10 (32.3%) in the control group had an irregular pattern (p = 0.40). In addition, in the two groups, the number of women with regular menstruation was higher than those with an irregular one. The volume of menstrual bleeding and the number of menstrual days before and after the intervention did not show a statistically significant difference between the two groups (Table II).

As illustrated in Table II, the incidence of Asherman's syndrome in the case group receiving hyaluronic acid was significantly lower than the controls. In the case group, only four women had poor adhesion at the end of the study, while the rest of them were free of any adhesions in the uterine cavity (Asherman's syndrome). In the control group, however, only 19 were free of intrauterine adhesions and 12 had mild symptoms. None of the individuals in the case group reported any side effects of using hyaluronic acid (such as allergic reaction).

**Table 1:** Comparison of demographic characteristics in the two groups


**Variables**		**Case group (n = 34)**	**Control group (n = 31)**	**P-value**
Age (Yr)*		33.50 ± 5.01	34.00 ± 5.36	0.699
Gravidity**		1.83 ± 2.27 (3${}^{\#}$)	1.80 ± 1.94 (4${}^{\#}$)	0.787
Live birth*		1.17 ± 0.39	1.25±0.45	0.633
Abortion*		2.50 ± 1.31	2.00±0.50	0.294
Stillbirth*		2.83 ± 1.72	1.80 ± 1.23	0.182
Menstrual pattern**				
	**Regular**	21 (61.76%)	21 (67.74%)	
	**Irregular**	13 (38.24%)	10 (32.26%)	<brow>-2</erow> 0.79
*Data presented as Mean ± SD, Student's *t* test, **Data presented as n (%), Chi-square test, ${}^{\#}$Interquartile range				

**Table 2:** Comparison of the number of days, the volume of menstrual bleeding, and the incidence of Asherman's syndrome in the two study groups


**Variables**		**Case group (n = 34)**	**Control group (n = 31)**	**P-value**
Menstrual bleeding pattern before the intervention*				
	**Hypomenorrhea**	5 (14.70)	3 (9.68)	
	**Normal menstruation**	23 (67.65)	22 (70.97)	
	**Hypermenorrhea**	6 (17.65)	6 (19.35)	<brow>-3</erow> 0.825${}^{a}$
Menstrual bleeding pattern after the intervention*				
	**Hypomenorrhea**	5 (14.70)	3 (9.68)	
	**Normal menstruation**	23 (67.65)	22 (70.97)	
	**Hypermenorrhea**	6 (17.65)	6 (19.35)	<brow>-3</erow> 0.825${}^{a}$
Days of the menstrual cycle**				
	**Before intervention**	6 ± 0.92	6.06 ± 1.06	0.794${}^{b}$
	**After intervention**	5.97 ± 0.94	6.06 ± 1.06	0.706${}^{b}$
Asherman's syndrome*				
	**No intrauterine adhesions**	30 (88.2)	19 (61.3)	
	**Poor intrauterine adhesions**	4 (11.8)	12 (38.7)	<brow>-2</erow> 0.012${}^{a}$
*Data presented as n (%). **Data presented as Mean ± SD. ${}^{a}$Chi-square test, ${}^{b}$Student's *t* test, Hypomenorrhea: < 20 ccs vaginal bleeding in one menstrual cycle, Normal menstruation: 20-80 cc of vaginal bleeding in one menstrual cycle, and Hypermenorrhea: > 80 ccs of vaginal bleeding in one menstrual cycle				

## 4. Discussion 

Uterine septum is one of the most common congenital abnormalities in women, associated with obstetric and fertility problems (17). In this study, the effect of hyaluronic acid intrauterine injection on the prevention of Asherman's syndrome in 65 women undergoing uterine septum resection was investigated. Evidenced by the results, intrauterine injection of hyaluronic acid reduced the incidence of Asherman's syndrome in the case group than the controls with no reported complications. Acunzo and co-workers in a randomized controlled trial identified that hyaluronic acid reduces the development of intrauterine adhesions following surgery and is likely to be associated with a reduction in severe adhesions (15). Hokker and colleagues also assessed the effect of hyaluronic acid on the prevention of Asherman's syndrome after dilatation and curettage (D & C). Their results revealed that the use of hyaluronic acid after D&C for abortion in women with at least one previous history of D&C reduces the incidence and severity of uterine adhesions but fails to eliminate the process of adhesion formation (18). Our study too, in line with this, indicated the significant effect of hyaluronic acid in reducing uterine adhesions. In a study by Guida and others performed on 130 patients in two groups (n = 65/each), the significant effect of hyaluronic acid after a three-month follow-up was observed in the intervention group. They described hyaluronic acid as a suitable and absorbable substance that provides an effective and safe strategy for improving women's health by reducing the need for re-intervention after hysteroscopic surgery (19). The two-month follow-up of the present study also confirms this claim. Furthermore, Mais and colleagues in their study on the reduction of postoperative adhesions with an injection of the hyaluronic gel after myomectomy with the laparoscopic method, being in line with the findings of the present study, reported that this method reduces the rate of adhesion and is safe and harmless (14).

In a randomized clinical trial on 84 women, the effect of hyaluronic acid was examined after Asherman's syndrome treatment. A follow-up hysteroscopy, three months after the surgery showed a significant reduction in intrauterine adhesions subsequent to the hyaluronic acid injection (14%) compared with the control group (32%) (20). Our study also demonstrated a significant effect similar to the case group. Researchers also believe that hyaluronic acid can be helpful in preventing the side effects of traditional anti-adhesives such as IUDs or foley catheters (21, 22). The results of the present study as well as several similar investigations reveal that the use of hyaluronic acid injected into the uterine cavity bears the potential to significantly prevent Asherman's syndrome in cases of endometrial damage.

## 5. Conclusion

Intrauterine injection of hyaluronic acid in women under uterine septum resection can significantly reduce the risk of Asherman's syndrome and is a safe method for the prevention of Asherman's syndrome.

##  Conflict of Interest

The authors declare no conflict of interest with regard to the present study.

## References

[1] Salazar CA, Isaacson K, Morris S. A comprehensive review of Asherman's syndrome: Causes, symptoms and treatment options. *Curr Opin Obstet Gynecol* 2017; 29: 249–256. 10.1097/GCO.0000000000000378 28582327

[2] Dreisler E, Kjer JJ. Asherman's syndrome: Current perspectives on diagnosis and management. *Int J Women's Health *2019; 11: 191–198. 10.2147/IJWH.S165474 PMC6430995 30936754

[3] Cenksoy PO, Ficicioglu C, Yesiladali M, Kizilkale O. The diagnosis and management of Asherman's syndrome developed after cesarean section and reproductive outcome. *Case Rep Obstet Gynecol* 2013; 2013: 450658. 10.1155/2013/450658 PMC3690225 23840987

[4] Aghajanova L, Cedars MI, Huddleston HG. Platelet-rich plasma in the management of Asherman syndrome: Case report. *J Assist Reprod Genet* 2018; 35: 771–775. 10.1007/s10815-018-1135-3 PMC5984883 29455274

[5] March CM. Management of Asherman's syndrome. *Reprod BioMed Online* 2011; 23: 63–76. 10.1016/j.rbmo.2010.11.018 21549641

[6] Papakonstantinou E, Roth M, Karakiulakis G. Hyaluronic acid: A key molecule in skin aging. *Dermatoendocrinol* 2012; 4: 253–258. 10.4161/derm.21923 PMC3583886 23467280

[7] Bukhari SNA, Roswandi NL, Waqas M, Habib H, Hussain F, Khan Sh, et al. Hyaluronic acid, a promising skin rejuvenating biomedicine: A review of recent updates and pre-clinical and clinical investigations on cosmetic and nutricosmetic effects. *Int J Biol Macromol* 2018; 120: 1682–1695. 10.1016/j.ijbiomac.2018.09.188 30287361

[8] Fei Zh, Xin X, Fei H, Yuechong C. Meta-analysis of the use of hyaluronic acid gel to prevent intrauterine adhesions after miscarriage. *Eur J Obstet Gynecol Reprod Biol* 2020; 244: 1–4. 10.1016/j.ejogrb.2019.10.018 31731019

[9] Conforti A, Alviggi C, Mollo A, De Placido G, Magos A. The management of Asherman syndrome: A review of literature. *Reprod Biol Endocrinol* 2013; 11: 118. 10.1186/1477-7827-11-118 PMC3880005 24373209

[10] Huberlant S, Fernandez H, Vieille P, Khrouf M, Ulrich D, Detayrac R, et al. Application of a hyaluronic acid gel after intrauterine surgery may improve spontaneous fertility: A randomized controlled trial in New Zealand White rabbits. *PloS One *2015; 10: e0125610. 10.1371/journal.pone.0125610 PMC4427444 25961307

[11] Krajčovičová R, Hudečk R, Ventruba P, Surgentová K. The role of hyaluronan in Asherman's syndrome therapy. *J Gynecol Surg* 2015; 31: 250–254.

[12] Liu H, Xu Y, Yi N, Yi W. Efficacy and safety of hyaluronic acid gel for the prevention of intrauterine adhesion: A meta-analysis of randomized clinical trials. *Gynecol Obstet Invest *2018; 83: 227–233. 10.1159/000486674 29514160

[13] Molotkov AS, Popov EN, Sudakov DS, Aivazyan TA, Alexandrova LA, Dymarskaya YR. Experience of intrauterine application of anti-adhesive gel based on hyaluronic acid in the prevention of Asherman's syndrome in patients with the pathology of the uterine cavity and severe forms of endometriosis. *J Obstet Women's Dis* 2017; 66: 12–19.

[14] Mais V, Bracco GL, Litta P, Gargiulo T, Melis GB. Reduction of postoperative adhesions with an auto-crosslinked hyaluronan gel in gynaecological laparoscopic surgery: A blinded, controlled, randomized, multicentre study. *Hum Reprod* 2006; 21: 1248–1254. 10.1093/humrep/dei488 16439505

[15] Acunzo G, Guida M, Pellicano M, Tommaselli GA, Di Spiezio Sardo A, Bifulco G, et al. Effectiveness of auto-cross-linked hyaluronic acid gel in the prevention of intrauterine adhesions after hysteroscopic adhesiolysis: A prospective, randomized, controlled study. *Hum Reprod* 2003; 18: 1918–1921. 10.1093/humrep/deg368 12923149

[16] Esmaeilzadeh S, Agajani Delavar M, Ghanbari Andarieh M. Reproductive outcome following hysteroscopic treatment of uterine septum. *Mater Sociomed* 2014; 26: 366–371. 10.5455/msm.2014.26.366-371 PMC4314157 25685079

[17] Practice Committee of the American Society for Reproductive Medicine. Uterine septum: A guideline. *Fertil Steril* 2016; 106: 530–540. 10.1016/j.fertnstert.2016.05.014 27235766

[18] Hooker AB, de Leeuw R, van de Ven PM, Bakkum EA, Thurkow AL, Vogel NEA, et al. Prevalence of intrauterine adhesions after the application of hyaluronic acid gel after dilatation and curettage in women with at least one previous curettage: Short-term outcomes of a multicenter, prospective randomized controlled trial. *Fertil Steril* 2017; 107: 1223–1231. 10.1016/j.fertnstert.2017.02.113 28390688

[19] Guida M, Acunzo G, Di Spiezio Sardo A, Bifulco G, Piccoli R, Pellicano M, et al. Effectiveness of auto-crosslinked hyaluronic acid gel in the prevention of intrauterine adhesions after hysteroscopic surgery: A prospective, randomized, controlled study. *Hum Reprod* 2004; 19: 1461–1464. 10.1093/humrep/deh238 15105384

[20] Tsapanos VS, Stathopoulou LP, Papathanassopoulou VS, Tzingounis VA. The role of Seprafilm bioresorbable membrane in the prevention and therapy of endometrial synechiae. *J Biomed Mater Res* 2002; 63: 10–14. 10.1002/jbm.10040 11787023

[21] Salma U, Xue M, Sheikh A, Sayed A, Xu D. Efficacy of intrauterine device in the treatment of intrauterine adhesions. *BioMed Res Int* 2014; 2014: 589296. 10.1155/2014/589296 PMC4165200 25254212

[22] Lin X, Wei M, Li TC, Huang Q, Huang D, Zhou F, et al. A comparison of intrauterine balloon, intrauterine contraceptive device and hyaluronic acid gel in the prevention of adhesion reformation following hysteroscopic surgery for Asherman syndrome: A cohort study. *Eur J Obstet Gynecol Reprod Biol* 2013; 170: 512–516. 10.1016/j.ejogrb.2013.07.018 23932377

